# Supplementary material for: Circulating human anti nucleolus antibody (ANCAb) and biochemical parameters in type 2 diabetic patients with and without complications
Source: PLoS One. 2020 Aug 17;15(8):e0237109. doi: 10.1371/journal.pone.0237109 (PMC7430723; doi:10.1371/journal.pone.0237109)
Supplement: S1 File — (PDF) [file pone.0237109.s001.pdf]

Human Anti Nucleolus Antibody

(ANCAb) Elisa kit (Competitive ELISA)

96 Tests

Catalog Number: MBS7228076

Store all reagents at 2-8°C

Valid Period: six months

For samples:
Serum, plasma, cell culture supernatants, body fluid and tissue homogenate

FOR RESEARCH USE ONLY!

NOT FOR THERAPEUTIC OR DIAGNOSTIC APPLICATIONS!

PLEASE READ THROUGH ENTIRE PROCEDURE BEFORE BEGINNING!

MyBioSource.com

FOR RESEARCH USE ONLY. NOT FOR USE IN DIAGNOSTIC PROCEDURES

Table of Contents

1. INTENDED USE 3
2. PRINCIPLE OF THE ASSAY 3
3. MATERIALS..4
4. SPECIMEN COLLECTION AND STORAGE 5
5. MATERIALS AND EQUIPMENT REQUIRED BUT NOT SUPPLIED...7
6. SAMPLE PREPARATION 7
7. REAGENTS PREPARATION...8
8. ASSAY PROCEDURE...9
9. CALCULATION OF RESULTS...10
10. CERTIFICATE OF ANALYSIS...12
11. SAFETY NOTES..13
12. QUALITY CONTROL 14
13. CONTACT US...16

12) Incomplete washing will adversely affect the test outcome. All washing must be performed with Wash Solution provided. All residual wash liquid must be drained from the wells by efficient aspiration or by decantation followed by tapping the plate forcefully on absorbent paper. Never insert absorbent paper directly into the wells.

13) Because TMB is light sensitive, avoid prolonged exposure to light. Also avoid contact between TMB and metal, otherwise color may develop.

MyBioSource.com

FOR RESEARCH USE ONLY. NOT FOR USE IN DIAGNOSTIC PROCEDURES

12. QUALITY CONTROL

1) It is recommended that all standards, controls and samples be run in duplicate. Standards and samples must be assayed at the same time.
2) The coefficient of determination of the standard curve should be ≥ 0.95 and the highest O.D. should be more than 1.0.
3) Cover or cap all kit components and store at 2-8° C when not in use.
4) Microtiter plates should be allowed to come to room temperature before opening the foil bags. Once the desired number of strips has been removed, immediately reseal the bag with desiccants and store at 2-8°C to maintain plate integrity.
5) Samples should be collected in pyrogen/endotoxin-free tubes.
6) Samples should be frozen if not analyzed shortly after collection. Avoid multiple freeze-thaw cycles of frozen samples. Thaw completely and mix well prior to analysis.
7) When possible, avoid use of badly hemolyzed or lipemic serum. If large amounts of particulate matter are present, centrifuge or filter prior to analysis.
8) When pipetting reagents, maintain a consistent order of addition from well-to-well. This ensures equal incubation times for all wells.
9) Do not mix or interchange different reagent lots from various kit lots.
10) Do not use reagents after the kit expiration date.
11) Read absorbance immediately after adding the stop solution.

1. INTENDED USE

This ANCAb ELISA kit is a 1.5 hour solid-phase ELISA designed for the quantitative determination of Human ANCAb. This ELISA kit for research use only, not for therapeutic or diagnostic applications!

2. PRINCIPLE OF THE ASSAY

ANCAb ELISA kit applies the competitive enzyme immunoassay technique utilizing Nucleolus antigen and an ANCAb-HRP conjugate. The assay sample and buffer are incubated together with ANCAb-HRP conjugate in pre-coated plate for one hour. After the incubation period, the wells are decanted and washed five times. The wells are then incubated with a substrate for HRP enzyme. The product of the enzyme-substrate reaction forms a blue colored complex. Finally, a stop solution is added to stop the reaction, which will then turn the solution yellow. The intensity of color is measured spectrophotometrically at 450nm in a microplate reader. The intensity of the color is inversely proportional to the ANCAb concentration since ANCAb from samples and ANCAb-HRP conjugate compete for the Nucleolus antigen binding site. Since the number of sites is limited, as more sites are occupied by ANCAb from the sample, fewer sites are left to bind ANCAb-HRP conjugate. A standard curve is plotted relating the intensity of the color (O.D.) to the concentration of standards. The ANCAb concentration in each sample is interpolated from this standard curve.

MyBioSource.com

FOR RESEARCH USE ONLY. NOT FOR USE IN DIAGNOSTIC PROCEDURES

3. MATERIALS

All reagents provided are stored at 2-8° C. Refer to the expiration date on the label.

	MATERIALS	SPECIFICATION	QUANTITY
1	MICROTITER PLATE	96 wells	stripwell
2	ENZYME CONJUGATE	6.0 mL	1 vial
3	STANDARD A	0 ng/mL	1 vial
4	STANDARD B	5.0 ng/mL	1 vial
5	STANDARD C	10 ng/mL	1 vial
6	STANDARD D	25 ng/mL	1 vial
7	STANDARD E	50 ng/mL	1 vial
8	STANDARD F	100 ng/mL	1 vial
9	SUBSTRATE A	6 mL	1 vial
10	SUBSTRATE B	6 mL	1 vial
11	STOP SOLUTION	6 mL	1 vial
12	WASH SOLUTION (100 x)	10 mL	1 vial
13	BALANCE SOLUTION	6 mL	1 vial
14	INSTRUCTION	1	

NOTE: The BALANCE SOLUTION is used only when the sample is **cell culture supernatants, body fluid and tissue homogenate**; if the sample is serum or plasma, then the BALANCE SOLUTION is a superfluous reagent.

cross-reactivity detection between ANCAb and all the analogues, therefore, cross reaction may still exist in some cases.

11. SAFETY NOTES

1) This kit contains small amount of 3, 3’, 5, 5’-Tetramethylbenzidine (TMB) in Substrate B. TMB is non-carcinogenic but it is hhazardous in case of skin contact, eye contact, ingestion and inhalation. In case of contact, rinse affected area with plenty of water.
2) The Stop Solution provided with this kit is an acid solution. Wear protective gloves, clothing, and face protection.
3) Care should be taken when handling the Standard because of the known and unknown effects of it.
4) Care should also be taken to avoid contact of skin or eyes with other kit reagents or specimens. In the case of contact, wash immediately with water.
5) Do not pipette by mouth.
6) Avoid generation of aerosols.
7) Waste must be disposed of in accordance with federal, state and local environmental control regulations.
8) All blood components and biological materials should be handled as potentially hazardous. Decontaminate and dispose specimens and all potentially contaminated materials as they could contain infectious agents. The preferred method of decontamination is autoclaving for a minimum of 1 hour a 121.5°C.

MyBioSource.com

NOTE:

1) Any variation in operator, pipetting and washing technique, incubation time or temperature, and kit age can cause variation in result. Each user should obtain their own standard curve.
2) If samples have been diluted, the concentration read from the standard curve must be multiplied by the dilution factor.
3) If specimens generate values higher than the highest standard, dilute the specimens and repeat the assay.

10. CERTIFICATE OF ANALYSIS

1) In the same lot CV%:<10
2) Different lot CV%: <10
3) Spike Recovery: 92-101%
4) Linearity:

	Range %
1:2	94 – 102
1:4	92 - 104
1:8	98 - 105
1:16	94 - 109

5) Sensitivity: The sensitivity in this assay is 1.0 ng/mL.
6) Specificity: This assay has high sensitivity and excellent specificity for detection of ANCAb. No significant cross-reactivity or interference between ANCAb and analogues was observed. **NOTE**: Limited by current skills and knowledge, it is impossible for us to complete the

The types of sample:	
Sample I:	serum or plasma
Sample II:	cell culture supernatants, body fluid and tissue homogenate

4. SPECIMEN COLLECTION AND STORAGE

Serum - Use a serum separator tube and allow samples to clot for 2 hours at room temperature or overnight at 2-8°C. Centrifuge at approximately 1000 × g (or 3000 rpm) for 15 minutes. Remove serum and assay immediately or aliquot and store samples at -20°C or -80°C.

Plasma - Collect plasma using EDTA or heparin as an anticoagulant. Centrifuge samples for 15 minutes at 1000 × g (or 3000 rpm) at 2 - 8°C within 30 minutes of collection. Assay immediately or aliquot and store samples at -20°C or -80°C.

Tissue homogenates - The preparation of tissue homogenates will vary depending upon tissue type. For this assay, tissues were rinsed in ice-cold PBS (0.02mol/L, pH 7.0-7.2) to remove excess blood thoroughly and weighed 300-500mg before homogenization. Minced the tissues to small pieces and homogenized them in 500ul of PBS with a glass homogenizer on ice. The resulting suspension was subjected to ultrasonication or to two freeze-thaw cycles to further break the cell membranes. After that, the homogenates were centrifugated for 15 minutes at 1500×g (or 5000 rpm).

MyBioSource.com

FOR RESEARCH USE ONLY. NOT FOR USE IN DIAGNOSTIC PROCEDURES

Remove the supernate and assay immediately or aliquot and store samples at -20°C or -80°C.

Cell lysates - Cells should be lysed according to the following directions.

1) Adherent cells should be detached with trypsin and then collected by centrifugation. Suspension cells can be collected by centrifugation directly.
2) Wash cells three times in PBS.
3) Cells were resuspended in PBS and subjected to ultrasonication for 3 times. Alternatively, freeze cells at -20 °C. Thaw cells with gentle mixing. Repeat the freeze/thaw cycle for 3 times.
4) Centrifuge at 1000×g (or 3000 rpm) for 15 minutes at 2-8 °C to remove cellular debris.
5) Assay immediately or store samples at -20°C or -80°C.

Cell culture supernatants and other body fluids - Centrifuge cell culture media at 1000 × g (or 3000 rpm) for 15 minutes to remove debris. Assay immediately or store samples at -20°C or -80°C.

NOTE:

1) Samples should be aliquoted and must be stored at -20°C (less than 3 months) or -80°C (less than 6 months) to avoid loss of bioactivity and contamination. If samples are to be run within 24 hours, they may be stored at 2- 8°C. Avoid repeated freeze-thaw cycles. Fresh samples without long time storage are recommended for the test. Otherwise, protein degradation and denaturalization may occur in those samples

1) The standard curve is used to determine the amount of samples.
2) First, average the duplicate readings for each standard and sample. All O.D. values are subtracted by the mean value of blank control before result interpretation. DO NOT subtract the O.D of standard zero.
3) Construct a standard curve by plotting the average O.D. for each standard on the vertical (Y) axis against the concentration on the horizontal (X) axis, and draw a best fit curve e using graph paper or statistical software to generate a four paramater logistic (4-PL) curve-fit or logit-log linear regression curve. An x-axis for the optical density and a y-axis for the concentration is also a choice. The data may be linearized by plotting the log of the concentrations versus the log of the O.D. and the best fit line can be determined by regression analysis.
4) Calculate the concentration of samples corresponding to the mean absorbance from the standard curve.
5) Standard curve for demonstration only.

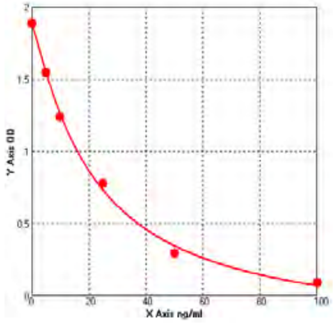

MyBioSource.com

FOR RESEARCH USE ONLY. NOT FOR USE IN DIAGNOSTIC PROCEDURES

4) Wash the microtiter plate using one of the specified methods indicated below:

a) Manual Washing: Remove incubation mixture by aspirating contents of the plate into a sink or proper waste container. Fill in each well completely with 1× wash solution, and then aspirate contents of the plate into a sink or proper waste container. Repeat this procedure five times for a total of FIVE washes. After washing, invert plate, and blot dry by hitting the plate onto absorbent paper or paper towels until no moisture appears. Note: Hold the sides of the plate frame firmly when washing the plate to assure that all strips remain securely in frame. Complete removal of liquid at each step is essential to good performance.

b) Automated Washing: Wash plate FIVE times with diluted wash solution (350-400 µL/well/wash) using an auto washer. After washing, dry the plate as above. It is recommended that the washer be set for a soaking time of 10 seconds and shaking time of 5 seconds between each wash.

5) Add 50 µL **Substrate A** and 50 µL **Substrate B** to each well including blank control well, subsequently. Cover and incubate for 10-15 minutes at 20-25°C. (Avoid sunlight).

6) Add 50 µL of **Stop Solution** to each well including blank control well. Mix well.

7) Determine the Optical Density (O.D.) at 450 nm using a microplate reader immediately.

9. CALCULATION OF RESULTS

2) Tissue or cell extraction samples prepared by chemical lysis buffer may cause unexpected ELISA results due to the impacts of certain chemicals.

3) Samples containing a visible precipitate must be clarified prior to use in the assay. Care should be taken to minimize hemolysis. Do not use grossly hemolyzed or lipemic specimens.

4) Do not use heat-treated specimens.

5. MATERIALS AND EQUIPMENT REQUIRED BUT NOT SUPPLIED

1) Precision pipettors and disposable tips to deliver 10-1000 µl. A multi-channel pipette is desirable for large assays.
2) 100 mL and 1 liter graduated cylinders.
3) Distilled or deionized water.
4) Tubes to prepare sample dilutions.
5) Absorbent paper.
6) Microplate reader cANCAble of measuring absorbance at 450 nm.
7) Centrifuge cANCAble of 3000 × g.
8) Microplate washer or washing bottle.
9) Incubator (37°C).
10) Data analysis and graphing software.

6. SAMPLE PREPARATION

1) We are only responsible for the kit itself, but not for the samples consumed during the assay. The user should calculate the

MyBioSource.com

FOR RESEARCH USE ONLY. NOT FOR USE IN DIAGNOSTIC PROCEDURES

possible amount of the samples used in the whole test. Please reserve sufficient amount of samples in advance.

2) Please predict the concentration before assaying. If values for these are not within the range of the standard curve, **users must determine the optimal sample dilutions for their particular experiments**. We suggest pre-experimenting with neat (undiluted) samples, 1:2 or 1:4 dilutions. Please avoid diluting your samples more than 1:10 as it would exceed the dilution limit set for this kit. If the expected concentration of the target is beyond the detection range of the kit, please contact technical support.

3) If the samples are not indicated in the manual, a preliminary experiment to determine the validity of the kit is necessary.

4) Owing to the possibility of mismatching between antigen from other resource and antibody used in our kits (e.g., antibody targets conformational epitope rather than linear epitope), some native or recombinant proteins from other manufacturers may not be recognized by our products.

5) Influenced by the factors including cell viability, cell number and also sampling time, samples from cell culture supernatant may not be detected by the kit.

7. REAGENTS PREPARATION

14) Bring all kit components and samples to room temperature (20-25 °C) before use.

15) Samples - Please predict the concentration before assaying. If concentrations are unknown or not within the detection range, a preliminary experiment is recommended to determine the optimal

dilution. PBS (pH 7.0-7.2) or 0.9% physiological saline can be used as dilution buffer.

16) Wash Solution - Dilute 10 mL of Wash Solution concentrate (100×) with 990 mL of deionized or distilled water to prepare 1000 mL of Wash Solution (1×). If crystals have formed in the concentrate, warm to room temperature and mix gently until the crystals have completely dissolved. The 1× wash solution is stable for 2 weeks at 2-8°C.

17) Do not dilute the other components which are ready- to-use.

8. ASSAY PROCEDURE

Please read Reagents Preparation before starting assay procedure. It is recommended that all Standards and Samples be assayed in duplicate. It is strongly recommended to do a preliminary experiment before measuring all samples.

1) Secure the desired numbers of coated wells in the holder then add 100 μL of **Standards (**Shake the bottle of each standard gently by hand and Pipette up and down the solution of standard for 3 times before adding**)** or **Samples** to the appropriate well. Add 100 μL of PBS (pH 7.0-7.2) in the blank control well.

2) Dispense 10 μL of **Balance Solution** into 100 μL samples only, mix well. **(NOTE**: This step is required when the sample is cell culture supernatants, body fluid and tissue homogenate; if the sample is serum or plasma, then this step should be skipped.)

3) Add 50 μL of **Conjugate** to each well (NOT blank control well). Mix well. Mixing well in this step is important. Cover and incubate the plate for 1 hour at 37°C.

MyBioSource.com

FOR RESEARCH USE ONLY. NOT FOR USE IN DIAGNOSTIC PROCEDURES
