# Supplementary material for: Circulating human anti nucleolus antibody (ANCAb) and biochemical parameters in type 2 diabetic patients with and without complications
Source: PLoS One. 2020 Aug 17;15(8):e0237109. doi: 10.1371/journal.pone.0237109 (PMC7430723; doi:10.1371/journal.pone.0237109)
Supplement: Attachment — Submitted filename: Response to Reviewers.docx [file pone.0237109.s003.docx]

PONE-D-20-08208
Circulating Human Anti Nucleolus Antibody (ANCAb) and Biochemical Parameters In Type 2 Diabetic Patients with and without Complications
PLOS ONE

Dear Mr. Abdulah,

We would appreciate receiving your revised manuscript by Jun 28 2020 11:59PM. When you are ready to submit your revision, log on to <https://www.editorialmanager.com/pone/> and select the 'Submissions Needing Revision' folder to locate your manuscript file.

To enhance the reproducibility of your results, we recommend that if applicable you deposit your laboratory protocols in [protocols.io](http://protocols.io), where a protocol can be assigned its own identifier (DOI) such that it can be cited independently in the future. For instructions see: <http://journals.plos.org/plosone/s/submission-guidelines#loc-laboratory-protocols>

Please include the following items when submitting your revised manuscript:

- A rebuttal letter that responds to each point raised by the academic editor and reviewer(s). This letter should be uploaded as separate file and labeled 'Response to Reviewers'.
- A marked-up copy of your manuscript that highlights changes made to the original version. This file should be uploaded as separate file and labeled 'Revised Manuscript with Track Changes'.
- An unmarked version of your revised paper without tracked changes. This file should be uploaded as separate file and labeled 'Manuscript'.

When submitting your revision, we need you to address these additional requirements.

1. Please ensure that your manuscript meets PLOS ONE's style requirements, including those for file naming. The PLOS ONE style templates can be found at <https://journals.plos.org/plosone/s/file?id=wjVg/PLOSOne_formatting_sample_main_body.pdf> and <https://journals.plos.org/plosone/s/file?id=ba62/PLOSOne_formatting_sample_title_authors_affiliations.pdf>

**Comment**:

2. We suggest you thoroughly copyedit your manuscript for language usage, spelling, and grammar. If you do not know anyone who can help you do this, you may wish to consider employing a professional scientific editing service.  
**Response**: The manuscript was send to SCREBENDI Language services. The certificate and its tracked changes was attached as well.

Upon resubmission, please provide the following:

- The name of the colleague or the details of the professional service that edited your manuscript
- A copy of your manuscript showing your changes by either highlighting them or using track changes (uploaded as a *supporting information* file)
- A clean copy of the edited manuscript (uploaded as the new *manuscript* file)

**Comment**:

3. In your Data Availability statement, you have not specified where the minimal data set underlying the results described in your manuscript can be found. PLOS defines a study's minimal data set as the underlying data used to reach the conclusions drawn in the manuscript and any additional data required to replicate the reported study findings in their entirety. All PLOS journals require that the minimal data set be made fully available. For more information about our data policy, please see <http://journals.plos.org/plosone/s/data-availability>.

**Response**: I responded the question. Please see the application.

**Comment**:

Upon re-submitting your revised manuscript, please upload your study’s minimal underlying data set as either Supporting Information files or to a stable, public repository and include the relevant URLs, DOIs, or accession numbers within your revised cover letter. For a list of acceptable repositories, please see <http://journals.plos.org/plosone/s/data-availability#loc-recommended-repositories>. Any potentially identifying patient information must be fully anonymized.

**Response**: I responded the question. Please see the application.

**Comment**:

Important: If there are ethical or legal restrictions to sharing your data publicly, please explain these restrictions in detail. Please see our guidelines for more information on what we consider unacceptable restrictions to publicly sharing data: <http://journals.plos.org/plosone/s/data-availability#loc-unacceptable-data-access-restrictions>. Note that it is not acceptable for the authors to be the sole named individuals responsible for ensuring data access.

**Response**: There is no restriction to share the data to the public. Just it would be applicable after acceptance.

We will update your Data Availability statement to reflect the information you provide in your cover letter.

4. Thank you for stating the following in the Acknowledgments Section of your manuscript:

'The study was funded by the authors only.'

**Comment**:

We note that you have provided funding information that is not currently declared in your Funding Statement. However, funding information should not appear in the Acknowledgments section or other areas of your manuscript. We will only publish funding information present in the Funding Statement section of the online submission form.

**Response**: The study was not funded by any organization. The statement was removed from the manuscript.

**Comment**:

Please remove any funding-related text from the manuscript and let us know how you would like to update your Funding Statement. Currently, your Funding Statement reads as follows:

'None'

5. Thank you for stating the following in your Competing Interests section:

'None'

**Response**: I removed the funding statement from the manuscript.

**Comment**:

Please complete your Competing Interests on the online submission form to state any Competing Interests. If you have no competing interests, please state "The authors have declared that no competing interests exist.", as detailed online in our guide for authors at <http://journals.plos.org/plosone/s/submit-now>

**Response**: I filled the form.

**Comment**:

 This information should be included in your cover letter; we will change the online submission form on your behalf.

**Response**: This information was added to the cover letter. Please see it in the attached file.

Please know it is PLOS ONE policy for corresponding authors to declare, on behalf of all authors, all potential competing interests for the purposes of transparency. PLOS defines a competing interest as anything that interferes with, or could reasonably be perceived as interfering with, the full and objective presentation, peer review, editorial decision-making, or publication of research or non-research articles submitted to one of the journals. Competing interests can be financial or non-financial, professional, or personal. Competing interests can arise in relationship to an organization or another person. Please follow this link to our website for more details on competing interests: <http://journals.plos.org/plosone/s/competing-interests>

**Comment**:

6. Please ensure that you refer to Figure 1 in your text as, if accepted, production will need this reference to link the reader to the figure.

**Response**: The figures were referenced to the text in the results. Please see the manuscript.

**Comment**:

7. Please include captions for your Supporting Information files at the end of your manuscript, and update any in-text citations to match accordingly. Please see our Supporting Information guidelines for more information: <http://journals.plos.org/plosone/s/supporting-information>.
Additional Editor Comments (if provided):

**Response**: We have not any supporting file for this manuscript.

**Reviewers' comments:**
Reviewer's Responses to Questions
**Comments to the Author**

**Comment**:

1. Is the manuscript technically sound, and do the data support the conclusions?

The manuscript must describe a technically sound piece of scientific research with data that supports the conclusions. Experiments must have been conducted rigorously, with appropriate controls, replication, and sample sizes. The conclusions must be drawn appropriately based on the data presented.

Reviewer #1: Partly

Reviewer #2: Partly

**Response**: We tried to make a connection between the results and conclusions. Please see the manuscript.

**Comment**:

2. Has the statistical analysis been performed appropriately and rigorously?

Reviewer #1: No

Reviewer #2: I Don't Know

**Response**: I checked the statistical extractions of the manuscript. Please see it.

**Comment**:

3. Have the authors made all data underlying the findings in their manuscript fully available?

The [PLOS Data policy](http://www.plosone.org/static/policies.action#sharing) requires authors to make all data underlying the findings described in their manuscript fully available without restriction, with rare exception (please refer to the Data Availability Statement in the manuscript PDF file). The data should be provided as part of the manuscript or its supporting information, or deposited to a public repository. For example, in addition to summary statistics, the data points behind means, medians and variance measures should be available. If there are restrictions on publicly sharing data—e.g. participant privacy or use of data from a third party—those must be specified.

Reviewer #1: No

Reviewer #2: No

**Response**: We uploaded the SPSS file for the review process if required.

**Comment**:

4. Is the manuscript presented in an intelligible fashion and written in standard English?

PLOS ONE does not copyedit accepted manuscripts, so the language in submitted articles must be clear, correct, and unambiguous. Any typographical or grammatical errors should be corrected at revision, so please note any specific errors here.

Reviewer #1: Yes

Reviewer #2: Yes

**Response**: Thanks.

**Comment**:

5. Review Comments to the Author

Please use the space provided to explain your answers to the questions above. You may also include additional comments for the author, including concerns about dual publication, research ethics, or publication ethics. (Please upload your review as an attachment if it exceeds 20,000 characters)

Reviewer #1: Deldar Morad et al. describe the prevalence and concentration of human anti-nucleolus autoantibodies (ANCAb) in a small cohort of individuals with diabetes mellitus type 2 (T2DM), respectively in subjects with microvascular complications (n=38) and without complications (n=43).

Though the topic is interesting per se, there are several severe systematic and design problems.

**Comment**:

- Why did the authors choose anti-nucleolus autoantibodies (ANCAb) as their target biomarker?

**Response**: The role of Anti-nuclear antibodies (ANA) has been investigated in few studies with controversial findings. We hypothesized that the autoimmunity could be developed within an anti-nucleolus rather than nuclear level. Therefore, we measured anti-nucleolus autoantibodies (ANCAb) between the patients with and without micro-complications in T2DM patients.

**Comment**:

What would be other autoimmune reactions in this group of individuals with T2DM? Picking one autoantibody is not enough to characterize a potential autoimmune component.

**Response**: It is right, but we had not sufficient financial resources to include other autoantibodies in the study.

**Comment**:

- Though the duration of T2DM is described to be similar, the definition of microvascular complications is very vague and several sub-entities are mixed-up in this group.

**Response**: The primary endpoint was the first record of one of the following 12 cardiovascular presentations in any of the
data sources: stable angina, unstable angina, myocardial infarction, unheralded coronary death, heart failure, transient ischaemic attack, ischaemic stroke, subarachnoid haemorrhage, intracerebral haemorrhage, peripheral arterial disease, abdominal aortic aneurysm, and a composite outcome consisted of cardioversion, ventricular arrhythmia, implantable cardioverter defibrillator, cardiac arrest, or sudden cardiac death (Reference: Shah, A.D., Langenberg, C., Rapsomaniki, E., Denaxas, S., Pujades-Rodriguez, M., Gale, C.P., Deanfield, J., Smeeth, L., Timmis, A. and Hemingway, H., 2015. Type 2 diabetes and incidence of cardiovascular diseases: a cohort study in 1· 9 million people. *The lancet Diabetes & endocrinology*, *3*(2), pp.105-113.

**Comment**:

- There is no clear procedure, how the individuals “without complications” have been examined.

**Response**: The patients who attended the out-patient clinic for medical check-up or receiving their regular medications were screened physically and clinically by the study internist. The study internist has weekly regular clinical duty in the out-patient clinic. The patients were screened for the possible complications of T2DM and consisted microvascular diseases, neuropathy, nephropathy, and retinopathy. The patients who were diagnosed to have one of the mentioned above complications following diabetes were categorized as T2DM patients with complication. The T2DM patients who did not have the complications were categorized as T2DM patients without complications. Please see the manuscript.

**Comment**:

Ocular tests only in patients with self-reported complaints is far from being the right screening method in this disease. Therefore, the assignment in the one or the other group might be misleading and explaining the lack of differences.

**Response**: We did not refer all patients to an ophthalmologist. We felt that it is not necessary to refer all patients to the ophthalmologist. Therefore, only patients who had a complaint on their visions during investigation were referred to the eye clinic.

**Comment**:

- Performing one assay of one company in these circumstances is not a suitable approach.

**Response**: We had limited financial resources to purchase the material assays of another company as well, since the study was not funded by any organization.

**Comment**:

Technical problems might have led to a number of zero values, as shown in Figure 1 – there is no description on assay performance and sensitivity/specificity, neither on false positive or false negative rate.

**Response**: We added the required information. Please see the manuscript.

**Comment**:

- The duration of T2DM in both groups (9.0 vs. 5.0 years; P=0.065) is very diverse, as seen in the p value – with 4 years from diagnosis and a potential longer undiagnosed duration before, these groups are not comparable

**Response**: I checked the diverse of the disease duration in a dot plot. They are not diverse. They have just some outliers in both groups. But, the outliers are not extreme. Please see the following graph.

**Comment**:

- As male and female individuals have very different risk factors for vascular diseases and autoimmunity, they should not be mixed up in groups. The more in the small groups in this study.

**Response**: Both groups have more females than males with the statistically comparable way (P=0.244). We did the comparison of ANCAb between males and females in Table 3. However, we could not include only males or females, because we could not obtain the target number of complicated patients within the planned time period. In that case, we needed longer time period to include the target number of patients into the study.

**Comment**:
- The conclusions are not supported by the data and analyses.

**Response**: I did revision in the conclusions. Please see the manuscript.

Reviewer #2: In this study the authors want to further elucidate the role of autoimmunity in Type 2 diabetes. To do that they investigated whether there are qualitative and quantitative differences in the levels of Human Anti Nucleolus Antibody (ANCAb) in sera of Type 2 diabetes patients with and without diabetes-related complications. The levels of ANCAb were determined by using a ELISA kit for the quantitative determination of ANCAb in serum. The authors did not find differences in reaction to ANCAb or of the amount of ANCAb in the sera of patients without diabetic complication compared to those with complications. There were also no differences in the comparison of specific complications (e.g. neuropathy) with no complications.

I think generally the results could match the criteria for publication but one major issue and some minors should be addressed before:
 **Comment**:

1) Methods:
The main question for me arises in regard to the performed ELISA and the results obtained by it. In the methods section the authors nicely report the patient selection and clinical evaluation. However the description of the Human Anti Nucleolus Antibody measurement is not sufficient in my point of view:
Please provide detailed protocol of how you performed the assay. Most of the results the authors report are in regard to “reaction to ANCAb”. I can’t find any description of what is actually meant by that or how that measurement was performed, or which cut-offs were used (“In the current study, any reaction of ANCAb with components of the body's healthy cells is was considered a reacted ANCAb.”).

**Response**: The required information was added to the methods section. Please see the manuscript.

**Comment**:

Can you give any references of other studies which used that assay? How are the results when a healthy control group is tested in regard to “any reaction of ANCAb with components of the body healthy cells”.

**Response**: With respect to the ANCAb, this is the first study that used ANCAb for the T2DM patients. However, about ANA, there are few studies; for example the following:

Grainger, D.J. and Bethell, H.W.L., 2002. High titres of serum antinuclear antibodies, mostly directed against nucleolar antigens, are associated with the presence of coronary atherosclerosis. *Annals of the rheumatic diseases*, *61*(2), pp.110-114.

Janahi, N.M., Santos, D., Blyth, C., Bakhiet, M. and Ellis, M., 2015. Diabetic peripheral neuropathy, is it an autoimmune disease?. *Immunology letters*, *168*(1), pp.73-79.

**Comment**:

Minor:
Introduction:
- In their introduction the authors briefly highlight some findings in regard to autoimmunity in diabetes type 1 and 2 and at the end focus on the possible role of anti-nuclear antibodies. Maybe you can provide any thoughts or rationale on why to test the specific subset of Anti Nucleolus Antibody.

**Response**: We found few investigation that examined the role of antinuclear antibodies (ANA) in development of complications in T2DM patients. We hypothesized that development of complications in T2DM patients may be searched in an anti- nucleolus level rather than nuclear one. Therefore, we decided to work on the anti- nucleolus layer rather than nuclear one.

 **Comment**:

- The authors state: “Although different studies suggested that antibodies may be present in the sera of T2DM patients with complications […]”
Please specify.
**Response**: We specified the studies. Please see the manuscript.

**Comment**:

- Please check the abbreviations. If an abbreviation is introduced it should be used from there on consistently (e.g. T2DM, ANCAb …)
**Response**: I checked and revised as appropriate.

**Comment**:

Methods:
- Section “Diagnosis and Measurement Criteria” : Provide units for HbA1c.

**Response**: It was provided. Please see the manuscript.

**Comment**:

- Section“ Human Anti Nucleolus Antibody measurement”:
“Serum ANCAb was performed […]” ◊ Sentence doesn’t make sense, please reframe.
“In the current study, any reaction of ANCAb with components of the body's healthy cells is was considered a reacted ANCAb” ◊ Check again (“is was”)

**Response**: They were revised as appropriate. Please see the manuscript.

**Comment**:

Results:
- At the end of the manuscript you provide Figure 1 A-D. However the figure is not mentioned in the text at all. Please also provide detailed figure legends.

**Response**: That is right, I did the reference to the text. Please see the manuscript.

**Comment**:

- In table 5 you provide the number of patients affected by several complications or combinations of complications. However some are missing e.g. the number of patients suffering from CVD-complication is not given and you didn’t test for differences between CVD-complications and without complications. Is there a reason why some of the complications have been left out or have not been tested?

**Response**: We had not sufficient number of patients with CVD in both group to make a comparison.

**Comment**:

- Paragraph 5 of the result section: “To have a better picture of the reaction to ANCAb in patients with different types of complications, the prevalence of ANCAb reaction in different categories of complications was presented in Table 4”.
◊ Table 4 shows the comparison between controlled and uncontrolled diabetes not the different categories of complications

**Response**: It was changed to the Table 5. Please see the manuscript.

 6. PLOS authors have the option to publish the peer review history of their article ([what does this mean?](https://journals.plos.org/plosone/s/editorial-and-peer-review-process#loc-peer-review-history)). If published, this will include your full peer review and any attached files.
If you choose “no”, your identity will remain anonymous but your review may still be made public.

**Do you want your identity to be public for this peer review?** For information about this choice, including consent withdrawal, please see our [Privacy Policy](https://www.plos.org/privacy-policy).

Reviewer #1: No

Reviewer #2: No

[NOTE: If reviewer comments were submitted as an attachment file, they will be attached to this email and accessible via the submission site. Please log into your account, locate the manuscript record, and check for the action link "View Attachments". If this link does not appear, there are no attachment files to be viewed.]

While revising your submission, please upload your figure files to the Preflight Analysis and Conversion Engine (PACE) digital diagnostic tool, <https://pacev2.apexcovantage.com/>. PACE helps ensure that figures meet PLOS requirements. To use PACE, you must first register as a user. Registration is free. Then, login and navigate to the UPLOAD tab, where you will find detailed instructions on how to use the tool. If you encounter any issues or have any questions when using PACE, please email us at [figures@plos.org](mailto:figures@plos.org). Please note that Supporting Information files do not need this step.
