# Supplementary material for: Circulating human anti nucleolus antibody (ANCAb) and biochemical parameters in type 2 diabetic patients with and without complications
Source: PLoS One. 2020 Aug 17;15(8):e0237109. doi: 10.1371/journal.pone.0237109 (PMC7430723; doi:10.1371/journal.pone.0237109)
Supplement: Attachment — Submitted filename: Response to reviewers.docx [file pone.0237109.s004.docx]

PONE-D-20-08208R1
Circulating Human Anti Nucleolus Antibody (ANCAb) and Biochemical Parameters In Type 2 Diabetic Patients with and without Complications
PLOS ONE

Dear Dr. Abdulah,

Please include the following items when submitting your revised manuscript:

- A rebuttal letter that responds to each point raised by the academic editor and reviewer(s). You should upload this letter as a separate file labeled 'Response to Reviewers'.
- A marked-up copy of your manuscript that highlights changes made to the original version. You should upload this as a separate file labeled 'Revised Manuscript with Track Changes'.
- An unmarked version of your revised paper without tracked changes. You should upload this as a separate file labeled 'Manuscript'.

Kind regards,

Andreas Zirlik, MD
Academic Editor
PLOS ONE

**6. Review Comments to the Author**

Please use the space provided to explain your answers to the questions above. You may also include additional comments for the author, including concerns about dual publication, research ethics, or publication ethics. (Please upload your review as an attachment if it exceeds 20,000 characters)

**Comment:** Reviewer #1: Some of the issues have been addressed, but several important points, like the ocular tests in ALL patients (which is mandatory for every DM subject and would only support the statistical outcome in terms of microvascular complications) seem to be not available.
In addition, the assay data are not convincing and not supportive for the author's conclusions.
Priority for publication is therefore low.

**Response**: As we mentioned previously, we did not perform the ocular tests for all patients. The patients were physically and clinically screened by the study internist for any possible complications. The patients who had vision complaints were referred to an ophthalmologist only. We honestly report that the ocular tests were not performed for all patients. We added into the limitations of the study as well.

Reviewer #2: In my opinion, the revised version of the manuscript provided by the authors has improved in quality but there are still some issues that need to be addressed:

**Comment:** 1) Can you give any references of other studies which used that assay? How are the results when a healthy control group is tested in regard to “any reaction of ANCAb with components of the body healthy cells”.

**Response**: We did not find other references that used this test. But, the following is the link of the test company with required information. We attached the catalogue of the company for this test into the application.

The link of the test of the company:

<https://www.mybiosource.com/human-elisa-kits/anti-nucleolus-antibody-ana/7228076>

Also, we found this test from another company as well. The following is the link of this test for the Human anti-nucleolus antibody and Mouse anti-nucleolus antibody test, respectively.

<https://www.biocompare.com/9956-Assay-Kit/2796027-Human-anti-nucleolus-antibody-ANA-ELISA-Kit/?pda=9956|2796027_0_0||11|ANA>

<https://www.biocompare.com/25138-Assay-Kit/13280700-Mouse-anti-nucleolus-antibody-ANA-ELISA-Kit/?pda=9956|13280700_0_0||27|ANA>

Concerning healthy controls, it was good to include the healthy controls in the study. However, we had financial restrictions for this study. Therefore, we added as a limitation into the study.

**Comment:**

Response: With respect to the ANCAb, this is the first study that used ANCAb for the T2DM patients. However, about ANA, there are few studies; for example the following: Grainger, D.J. and Bethell, H.W.L., 2002. High titres of serum antinuclear antibodies, mostly directed against nucleolar antigens, are associated with the presence of coronary atherosclerosis. Annals of the rheumatic diseases, 61(2), pp.110-114.
Janahi, N.M., Santos, D., Blyth, C., Bakhiet, M. and Ellis, M., 2015. Diabetic peripheral neuropathy, is it an autoimmune disease?. Immunology letters, 168(1), pp.73-79.
Comment: I understand this is the first study which tested for ANCAb in T2DM patients. My question was if there are data on ANCAb reaction in healthy controls. In their discussion the authors attribute the reported reactions to ANCAb to latent autoimmune diabetes in adults. I don't think this conclusion is valid if there is no comparison to a group of matched healthy controls. I would suggest to include a control group of matched healthy controls to see if there is a role of ANCAb reaction in T2DM patients at all.

**Response**: We could not include the healthy controls in this study due to financial resources. If we included the healthy controls, the sample size would be reduced more than this. We added this point to the study limitations as well. Please see the manuscript.

**Comment:** 2) One of the differences of the two groups was waist circumference with higher values in the non-complicated group (107.95 to 102.30). Also there was a trend to higher BMI in the non-complicated group. Since adipose tissue is known to contribute to secretion of autoimmune antibodies that potentially could disguise an estimated higher amount of ANCAb reaction in more complicated disease. Maybe the authors could add that in their discussion.

**Response**: We added the relevant information about this point. Please see the manuscript.

**Comment:**
3) I think many of the points reviewer 1 mentioned are valid (like verification by another method or looking for different autoantibodies) and if they cannot be resolved they should at least be discussed or added in the limitation section.

**Response**: We added this point to the limitation. Please see the manuscript.

**Comment:**
7. PLOS authors have the option to publish the peer review history of their article ([what does this mean?](https://journals.plos.org/plosone/s/editorial-and-peer-review-process#loc-peer-review-history)). If published, this will include your full peer review and any attached files.
**Response**: It is OK.
If you choose “no”, your identity will remain anonymous but your review may still be made public.
